# Use of Artificial Intelligence to Shorten the Behavioral Diagnosis of Autism

**DOI:** 10.1371/journal.pone.0043855

**Published:** 2012-08-27

**Authors:** Dennis P. Wall, Rebecca Dally, Rhiannon Luyster, Jae-Yoon Jung, Todd F. DeLuca

**Affiliations:** 1 Center for Biomedical Informatics, Harvard Medical School, Boston, Massachusetts, United States of America; 2 Department of Pathology, Beth Israel Deaconess Medical Center, Boston, Massachusetts, United States of America; 3 Laboratories of Cognitive Neuroscience, Boston Children’s Hospital, Boston, Massachusetts, United States of America; University of Glasgow, United Kingdom

## Abstract

The Autism Diagnostic Interview-Revised (ADI-R) is one of the most commonly used instruments for assisting in the behavioral diagnosis of autism. The exam consists of 93 questions that must be answered by a care provider within a focused session that often spans 2.5 hours. We used machine learning techniques to study the complete sets of answers to the ADI-R available at the Autism Genetic Research Exchange (AGRE) for 891 individuals diagnosed with autism and 75 individuals who did not meet the criteria for an autism diagnosis. Our analysis showed that 7 of the 93 items contained in the ADI-R were sufficient to classify autism with 99.9% statistical accuracy. We further tested the accuracy of this 7-question classifier against complete sets of answers from two independent sources, a collection of 1654 individuals with autism from the Simons Foundation and a collection of 322 individuals with autism from the Boston Autism Consortium. In both cases, our classifier performed with nearly 100% statistical accuracy, properly categorizing all but one of the individuals from these two resources who previously had been diagnosed with autism through the standard ADI-R. Our ability to measure specificity was limited by the small numbers of non-spectrum cases in the research data used, however, both real and simulated data demonstrated a range in specificity from 99% to 93.8%. With incidence rates rising, the capacity to diagnose autism quickly and effectively requires careful design of behavioral assessment methods. Ours is an initial attempt to retrospectively analyze large data repositories to derive an accurate, but significantly abbreviated approach that may be used for rapid detection and clinical prioritization of individuals likely to have an autism spectrum disorder. Such a tool could assist in streamlining the clinical diagnostic process overall, leading to faster screening and earlier treatment of individuals with autism.

## Introduction

Although autism has a considerable genetic component [1], it is currently diagnosed through behavior. The clinical practice of diagnosis has been formalized through instruments containing questions carefully designed to assess impairments in three developmental domains: communication and social interactions, restricted interests and activities, and stereotypical behaviors. One of the most widely adopted instruments is the Autism Diagnostic Interview – Revised (ADI-R) [2]. This examination contains 93 items targeted for individuals with a mental age 18 months or older. The exam is delivered in a clinical setting by a trained professional and can take up to 2.5 hours to complete. While the instrument is highly reliable, consistent across examiners [3]–[5], and results in a rich understanding of the individual suspected of having autism, its length can be prohibitive.

The practice of diagnosing autism varies widely in terms of standards and timeframes. Families may wait as long as 13 months between initial screening and diagnosis [6] and even longer if part of a minority population or lower socioeconomic status [7]. These delays directly translate into delays in the delivery of speech and behavioral therapies that have significant positive impacts on a child’s development, especially when delivered early [8], [9]. In addition, the recognition of the severity of the phenotype can vary significantly across clinicians performing formal diagnosis [10]. Thus a large percentage of the population is diagnosed after developmental windows in which behavioral therapy would have had maximal impact on future development and quality of life. The average age of diagnosis in the United States is 5.7 years and an estimated 27% remain undiagnosed at 8 years of age [11]. At these late stages in development, many of the opportunities to intervene with therapy have evaporated. A shortened and readily accessible test that can be delivered in advance or as part of a clinical visit could improve these statistics.

Significant attention has been paid to the design of abbreviated screening examinations that are meant to foster more rapid diagnosis, including the Autism Screening Questionnaire (ASQ, designed to discriminate between PDD and non-PDD diagnoses [12]), the Social Communication Questionnaire (SCQ) [13], and the Modified Checklist for Autism in Toddlers (MCHAT) [14]. However, most of these have been adopted for basic screening rather than formal diagnosis, and may often be used prior to administering the ADI-R or Autism Diagnostic Observation Schedule (ADOS) [15]. While some pediatricians conduct routine autism screenings during well-child visits, it has yet to become a universal practice [16] leaving much of the burden on the parent or care provider. Parents often hesitate to take immediate action without a clinical assessment and formal diagnosis, furthering delays in the treatment of the child through behavioral therapy or other means [8], [17]. An exam that preserves the reliability of the ADI-R but that can be administered in minutes rather than hours would enable more rapid clinical diagnosis, higher throughput, as well as timely and likely more impactful delivery of therapy.

A direct way to test whether an abbreviated ADI-R provides the same level of accuracy as the full examination is to look retrospectively at answers to the full ADI-R for a large set of individuals with autism. Many efforts to-date on shortening the behavioral diagnosis of autism have leveraged clinical experience and criteria established by the DSM-IV to prospectively design and test new instruments. However, as a valuable byproduct of the widespread adoption and use of ADI-R, we now have large digital repositories of item-level answers to each question coupled with the clinical diagnosis that can be mined to test this question directly. In the present study, we employed analytical strategies from the field of machine learning to retrospectively analyze the full ADI-R for over 800 individuals with autism, with our aim centered on significantly reducing the number of questions while preserving the classification given by the full ADI-R.

## Results

**Figure 1:**
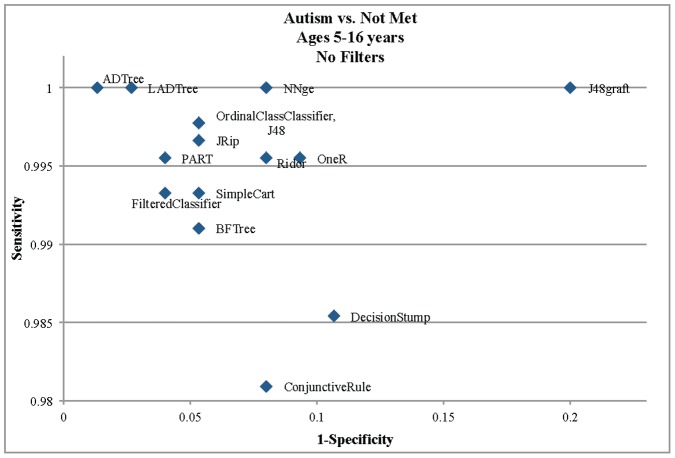
Performance of 15 machine learning algorithms evaluated for classifying autism cases and non-spectrum controls. Plot comparing 1-specificity and sensitivity for the 15 different machine learning algorithms used to construct classifiers from the 93-item Autism Diagnostic Interview-Revised (ADI-R) instrument from the Autism Genetic Resource Exchange (AGRE). The best performing algorithm was the alternating decision tree (ADTree), followed by LADTree, PART, and FilteredClassifier. Table 2 summarizes the 15 machine learning algorithms in more detail, and the elements contained in the ADTree classifier are listed in Table 3.

We began by downloading the complete set of ADI-R data from the Autism Genetic Resource Exchange (AGRE) (Table 1) for classifier training and testing. We then compared the performance of 15 different machine learning algorithms (Table 2) on the 93 item from the complete ADI-R and found that the Alternating Decision Tree (ADTree) performed best in terms of both sensitivity and specificity of classification, with perfect sensitivity of 1.0, a false positive rate (FPR) of 0.013, and overall accuracy of 99.90% (Figure 1). This classification algorithm creates a mapping from class instances to real numbers that is defined in terms of a set of base rules that when summed generate a real value prediction. The classification of an instance is the sign of the prediction. In our study, a negative sign corresponded to a classification of autism and a positive sign corresponded to a classification of non-spectrum. The ADTree classifier correctly classified all AGRE individuals previously labeled with a diagnosis of autism using the full ADI-R exam and misclassified only 1 control individual. The ADTree classifier contained only 7 questions from the 93 used in the analysis. These were ageabn, grplay5, conver5, peerpl5, gaze5, play5, and compsl5 (Table 3), and together represent a ∼93% reduction in the total number of elements overall.

**Table 1:** Summary of the data used for both construction and validation of the autism classifier.

	Classifier Data		Validation Data			
	AGRE		SSC		AC	
	Autism	Not Met	Autism	Not Met	Autism	Not Met
Sample Size	891	75	1654	5	322	12
Q1 (Age)	6.44	6.38	6.75	8.38	6.50	5.42
Median (Age)	8.06	9.24	8.75	9.75	8.50	9.50
Q3 (Age)	10.84	11.88	11.25	12.25	11.54	13.58
IQR (Age)	4.4	5.5	4.5	3.88	5.04	8.17

Full sets of answers to the Autism Diagnostic Instrument-Revised questionnaire were downloaded from the Autism Genetic Research Exchange (AGRE), the Simons Simplex Collection (SSC), and the Boston Autism Consortium (AC). The AGRE data were used for training, testing, and construction of the classifier. The SSC and AC data were used for independent validation of the resulting classifier. The table lists the total numbers of spectrum and non-spectrum individuals represented in each of the three data sets with a breakdown of age by quartiles.

**Table 2:** The 15 machine learning algorithms used to analyze the Autism Genetic Resource Exchange ADI-R data.

Classifier Name	Description	FPR	TPR	Accuracy
ADTree	An ADTree combines decision trees, voted decision trees, and voted decision stumps.This particular algorithm is based on boosting, which produces accurate predictionsby combining a series of “weak” learners that together, can classify accurately [29].	0.013	1.000	0.999
BFTree	The top node of the decision tree is the one that splitsthe data so that the maximum reduction of impurity (misclassified data) is achieved. This is called the “best” node,and it is expanded upon first (unlike in a C4.5 tree, for example, where nodes areexpanded upon according to depth-first) [30].	0.053	0.991	0.988
ConjunctiveRule	Within the ConjuctiveRule classifier is a conjunctive rule learner, which can predict forboth numeric and nominal class labels. A rule consists of a series of antecedents joinedby “AND”s [31].	0.080	0.981	0.976
DecisionStump	A DecisionStump classifier is a single-level decision tree with one node. The terminalnodes extend directly off of this node, so a classification is made based on asingle attribute [31].	0.107	0.985	0.978
FilteredClassifier	FilteredClassifier runs data through an arbitrary classifier after it’s been run through anarbitrary filter. Classifiers are built using training data, and in this case, the filter is alsobuilt based on the training data. This allows the user to skip the pre-processing stepsassociated with transforming the data [32].	0.040	0.993	0.991
J48	J48 is a Java implementation of the C4.5 algorithm; it generates either an unprunedor a pruned C4.5 decision tree. C4.5 uses the concept of information entropy tobuild trees from training data [33].	0.053	0.998	0.994
J48graft	This class generates a grafted C4.5 decision tree that can either be prunedor unpruned. Grafting adds nodes to already created decision trees to improveaccuracy [34].	0.200	1.000	0.984
JRip	This classifier is an optimized version of Incremental Reduced Error Pruning, andimplements a propositional learner, RIPPER (Repeated Incremental Pruning to ProduceError Reduction). It produces accurate and “readable” rules [35]	0.053	0.997	0.993
LADTree	LADTree produces a multi-class alternating decision tree. It has the capability tohave more than two class inputs. It uses the LogitBoost strategy, which performsadditive logistic regression [36]	0.027	1.000	0.998
NNge	Nearest neighbor algorithms define a distance function to separate classes. Usinggeneralized exemplars reduce the role of the distance function (relying too heavilyon the distance function can produce inaccurate results) by grouping classestogether [37].	0.080	1.000	0.994
OneR	This algorithm finds association rules. It finds the one attribute that classifiesinstances so as to reduce prediction errors [38].	0.093	0.996	0.989
OrdinalClassClassifier	This is a meta-classifier (meta-classifiers are likeclassifiers, but have added functionality) used totransform an ordinal class problem to a series of binary class problems [39].	0.053	0.998	0.994
PART	A set of rules is generated using the “divide-and-conquer” strategy. From here,all instances in the training data that are covered by this rule get removed and this processis repeated until no instances remain [40].	0.040	0.996	0.993
Ridor	This classifier is an implementation of a Ripple-Down Rule Learner. An example ofthis is when the classifier picks a default rule (based on the least weighted error),and creates exception cases stemming from this one [41]	0.080	0.996	0.990
SimpleCart	Classification and regression trees are used to construct prediction models for data.They are made by partitioning the data and fitting models to each partition [42].	0.053	0.993	0.990

These algorithms were deployed using the toolkit WEKA [28]. The false positive rate (FPR) and true positive rate (TPR) are provided together with overall accuracy. The Alternating Decision Tree (ADTree) performed with highest accuracy and was used for further analyses.

**Table 3:** The seven attributes used in the ADTree model.

ADI-R question	Abbreviation	Description
29	compsl5	Comprehension of simple language: answer most abnormal between 4 and 5
35	conver5	Reciprocal conversation (within subject’s level of language): answer if ever (when verbal)
48	play5	Imaginative play: answer most abnormal between 4 and 5
49	peerpl5	Imaginative play with peers: answer most abnormal between 4 and 5
50	gaze5	Direct gaze: answer most abnormal between 4 and 5
64	grplay5	Group play with peers: answer most abnormal between 4 and 5
86	ageabn	Age when abnormality first evident

Listed are the number corresponding to the question in the full ADI-R instrument, the question code used by Autism Genetic Research Exchange (AGRE) and a brief description of the question.

**Figure 2:**
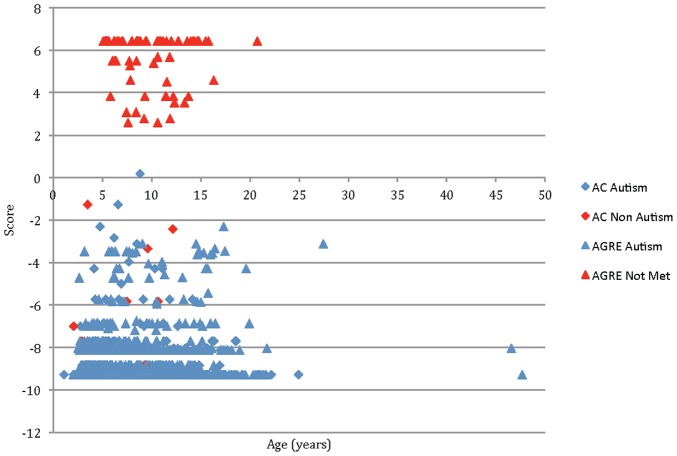
Decision tree scores and classification of cases with and without a diagnosis of autism. The Alternating Decision Tree (ADTree) scores of individuals in the both the AC and AGRE data sets versus their age in years. A majority of the ADTree scores were clustered towards greater magnitudes according to their respective classifications, regardless of age.

The 7 questions formed the elements of a decision tree through which the classifications of autism and non-spectrum were derived. Three questions appeared more than once in the tree (ageabn, play5, and peerpl5); each question either increased or decreased a sum score called the ADTree score. A negative score resulted in a classification of autism and a positive score yielded the classification of non-spectrum. The amplitude of the score provided a measure of confidence in classification outcome, with larger absolute values indicating higher confidence overall. In our study, the vast majority of the scores were near or at the maximum for both the case and control classes, with comparably few individuals classified with intermediate or low confidence values (Figure 2) indicating that the predictions made by the classifier were robust and well supported.

To independently validate our 7-question classifier, we used completed ADI-R score sheets from two repositories, the Simons Foundation (SSC) and the Boston Autism consortium (AC) (Table 1). The classifier performed with high accuracy on both the SSC and AC data sets. All individuals in the SSC previously diagnosed with autism were accurately classified as having autism by the classifier. In the AC, the classifier accurately classified 321 of the 322 cases with autism (99.7% accuracy). Interestingly, the single misclassified individual from AC was assigned a low-confidence ADTree score of 0.179 casting possible doubt on the classification and suggesting the potential that a further behavioral assessment of this individual could result in a non-spectrum diagnosis.

We then examined the classification performance on the few non-spectrum individuals present in the SSC and AC data collections. Three of the 5 SSC subjects were accurately classified, and 6 of the 12 AC subjects were accurately classified. Further inspection of these 8 misclassified controls revealed possible autism spectrum behaviors. Five had a previous diagnosis prior to recruitment to the study (2 with Asperger’s Syndrome and 3 with PDD-NOS) and all 8 were diagnosed on the autism spectrum by an alternative diagnostic instrument, the Autism Diagnostic Observation Schedule (ADOS) [15].

Given the large imbalance in numbers of autism cases and non-spectrum controls and the potential biases that such class imbalance could have on the outcome of the classifier, we elected to simulate 1000 non-spectrum controls by random sampling from the pool of observed answers given by 92 non-spectrum individuals. We then tested the performance of the 7-question classifier against this population of simulated data. The classifier performed with 99.9% specificity on these simulated controls, misclassifying only one. To broaden the distribution beyond that represented in the 92 observed samples, we also simulated 1000 new controls by creating random permutations of the 93 questions contained in the full ADI-R that would correspond to a non-spectrum diagnosis using the ADI-R algorithm. We ensured that these simulated data would fall close to but below established cutoffs for social interaction, communication, and repetitive behaviors. The classifier incorrectly categorized 62 of the 1000 controls simulated using this larger distribution of ADI-R data, corresponding to a specificity of 93.8%.

Given the importance of diagnosis at early ages, we also tested the accuracy of our classifier on the collection of answers from children diagnosed at ages under 5. Although 5 of the 7 questions in the classifier probe for the most abnormal behavior between 4 and 5 years of age, we speculated that the answers to those questions with the “current” behavior would be equally accurate and allow expansion to younger children. Because the SSC restricted case recruitment to ages 4 and older and we could use only the AGRE and AC datasets, which contained sufficient numbers of children below age 5, to test our hypothesis. For this analysis, we had a total of 1589 individuals previously listed as having autism in either AGRE or AC and 88 individuals flagged as not meeting the criteria for an autism diagnosis. All but 1 of the children with autism were correctly categorized by our classifier, a near perfect accuracy of 99.9%, and 12 of the 88 controls were misclassified as having autism, corresponding to an 86% accuracy. As in the validation steps above, all 12 of these individuals had a conflicting ADOS categorization, suggesting the possibility that additional inspection and behavioral analysis may reveal characteristics consistent with an autism diagnosis.

## Discussion

Current practices for the behavioral diagnosis of autism are highly effective but also prohibitively time consuming. A gold standard in the field is the Autism Diagnostic Interview-Revised (ADI-R), a 93-item exam that yields high inter-interviewer reliability and accuracy. In the present study, we used machine learning techniques to test whether the accuracy of the full ADI-R could be achieved with a significantly shorter exam. Our analysis found a small subset of 7 elements targeting social, communication, and behavioral abilities to be 99.97% as effective as the full ADI-R algorithm for classification of 2867 cases with autism drawn from three separate repositories. This represents 93% fewer questions than the full ADI-R exam and 84% fewer questions than contained in the ADI-R algorithm itself.

Our analysis used machine learning techniques to analyze previous collections of data from individuals with autism. In our case, several alternative machine learning strategies yielded classifiers with high accuracy and low rates of false positives. The top performing ADTree algorithm proved most valuable for classification as well as for measuring classification confidence, with a nearly 100% accuracy in the diagnosis of cases with autism. The ADTree algorithm resulted in a decision-tree classifier that could be converted into a behavioral algorithm for deployment in screening and/or diagnostic settings. In addition, the classifier generated a score that provided an empirical measure of confidence in the classification and that could be used to flag borderline cases likely warranting closer inspection and further behavioral assessment. In our case, a small number of controls were misclassified, but many of these classifications were given a low confidence score suggesting that further screening and additional diagnostic evaluation might reveal problems with the initial diagnoses.

The brevity of this machine learning classifier and its apparent accuracy for classification of subjects both on and off the spectrum suggests the possibility that it could be of value in early, rapid detection of autism, a potential shared by and possibly combinable with recent work on observation-based detection of autism [18]. An approach such as the one here could require less specialty training for administration and delivery and enable a wider reach to the population at risk, particularly if delivered through mobilized channels (internet and handheld devices) and used for prioritization of cases for subsequent clinical evaluation and diagnosis. However, work has shown performance problems of rapid assessment tools like the Social Responsiveness Scale and Social Communication Questionnaire [13] especially with differentiation of autism spectrum cases from other neurodevelopmental conditions and learning delays [19]–[21], as well as problems with agreement in the clinical diagnosis even when full ADI-R and other instruments are used in the diagnostic process [10]. Future work must therefore involve further exploration of the clinical potentials of this approach through prospective studies, being careful to consider the additional limitations covered in the section below.

### Limitations

Our study was limited by the content of existing repositories, and as consequence, we had a small number of matched controls for construction and validation of the classifier. In a prospective design for a study like ours, we would attempt to equalize the numbers of cases and controls for optimal calculations of sensitivity and specificity of the classifier. Nevertheless, the clear demarcation between cases and controls found with our existing data (Figure 2) provided some confidence that our classifier would scale to a larger population with equal or similar accuracy. In addition, our classifier performed with reasonable accuracy on two sets of simulated controls. Although the first set of simulated data were bounded by the empirical distribution of answers given by the true control individuals, that empirical distribution covered a space of answers likely to be provided by prospectively recruited controls. The second set explored permutations of the full ADI-R that would meet cutoff requirements for an ASD diagnosis in the core domains of social interaction, communication, and repetitive behavior, and resulted in a decreased specificity of 93%. Going forward, we hope to expand our validation via prospective studies that enable the inclusion of new ADI-R data from both individuals with autism as well as non-spectrum individuals with neurodevelopmental delays.

The data used also contained an abundance of older children, with highest density between ages of 5 and 17, potentially making the resulting classifier biased against effective assessment of younger children. However, we were able to show near perfect classification accuracy for children 4 years of age and younger, with the youngest individual being 13 months (Figure 2). Given that the sample sizes of younger children was small, we anticipate that a larger sample will provide greater resolution and a larger set of training data to develop and test if a new classifier can achieve greater accuracy than the one generated here.

Finally, since our classifier was trained only on individuals with or without classic autism it was not trained to pinpoint other diagnoses along the autism spectrum including Asperger and Pervasive Developmental Disorder-Not Otherwise Specified (PDD-NOS). This was a byproduct of the data available to us at the time of study, which did not have sufficient granularity to test whether the classifier could be utilized for more fine-grained diagnoses. Either a large sample of ADI-R data from a range of ASDs, a series of prospective clinical studies, and also potentially instrument administration via social networking and web-based technologies (for example, like the one we have launched at http://autworks.hms.harvard.edu/community/survey), would enable us to measure the performance of our classifier outside of classic autism, and would also enable retraining of the classifier should the performance decrease.

### Conclusions

Currently, the diagnosis of autism is through behavioral exams and questionnaires that require considerable time investment on the part of parents and clinicians. Here we attempted to reduce one of the most commonly used instruments for behavioral diagnosis, the Autism Diagnostic Interview-Revised (ADI-R) to begin addressing this time burden. Deploying a variety of machine learning algorithms, we found one, the Alternating Decision Tree (ADTree), to have high sensitivity and specificity in the classification of individuals with autism from controls The ADTree classifier consisted of only 7 questions, 93% fewer than the full ADI-R, and performed with greater than 99% accuracy when applied to independent populations of individuals with autism, misclassifying only one out of the 1962 cases used for validation. The classifier also performed with equally high accuracy on children under 4 and as young as 13 months, indicating its applicability to a younger population of children with autism. Given the dramatic reduction in numbers of questions without appreciable loss in accuracy, our findings may represent an important step towards making the diagnosis of autism a faster process that enables delivery of therapy at earlier and more impactful stages of child development.

## Methods

### Ethics Statement

Our study (number: M18096-101) has been evaluated by the Harvard Medical School Institutional Review Board and identified as not involving human subjects as defined under 45CFR46.102 (f) and as meeting the conditions regarding coded biological specimens or data. As such, (a) the specimens/data were not collected specifically for the research through an interaction or intervention with a living person, and (b) the investigators cannot “readily ascertain” the identity of the individual who provided the specimen/data to whom any code pertains. The Harvard Medical School Institutional Review Board determined the study to be exempt.

### Constructing a Classifier

For constructing a classifier, we used phenotype data from the Autism Genetic Resource Exchange [22] (AGRE) repository of families with at least one child with autism. Specifically, we used the answers to the 93 questions and sub-questions in the 2003 version of ADI-R. We restricted our initial analysis to children with a diagnosis of autism from the categories autism, broad spectrum, and “not quite autism”. Having one of these classifications was determined by the AGRE “affected status” algorithms, which used the domain scores from the ADI-R to evaluate the individuals. The autism classification used by AGRE follows the validated algorithm created by the authors of the ADI-R [2]. If a child who took the ADI-R did not meet any of these classification criteria, he or she was deemed non-spectrum, and was used as a control in our study. We also restricted our machine learning classification steps to subjects with and without an autism diagnosis who were 5 years of age or older and under the age of 17 years of age as the majority of data were from within this age range, thereby providing the most uniform collection of answers to the ADI-R and the most complete matrix of data for machine learning. These steps resulted in 891 individuals with a classification of autism and 75 with a classification of not met (Table 1). The answers to the 93 multiple-choice questions on the ADI-R were encoded as discrete numbers between 0–3. A score of 0 was given when “behavior of the type specified in the coding was not present.” A score of 1 was given when “behavior of the type specified was present in an abnormal form, but not sufficiently severe or frequent to meet the criteria for a 2.” A score of 2 indicated “definite abnormal behavior” meeting the criteria specified, and a score of 3 was reserved for “extreme severity” of the specified behavior, although scores of 3 were converted to 2 during scoring and classification. There were also scores of 7 (“definite abnormality in the general area of the coding, but not of the type specified”), 8 (“not applicable”), and 9 (“not known or not asked”) all of which were converted to 0 when conducting scoring and classification. The answers, converted to the values appropriate for scoring, were formatted into a matrix containing the cases, controls, and diagnostic labels.

Using this matrix we conducted a series of machine learning analyses to construct a classifier from the 93 ADI-R questions in order to distinguish individuals classified with autism from those deemed non-spectrum. We compared the performance of 15 machine learning algorithms (Table 2) that have been demonstrated to be of value for the learning and classification problem presented here. For each algorithm, we performed 10-fold cross validation – using 90% of the data for training and the remaining 10% for testing – to build and assess the accuracy of the resulting classifier. Such cross-validation has been shown to perform optimally for structured, labeled data while reducing bias in the resulting classifier [23] and was therefore best suited to our learning tasks. For each of the 15 classifiers, we measured the false positive rate (FPR), true positive rate (TPR), as well as the overall accuracy. We then plotted the specificity (FPR) against sensitivity (TPR) to visualize the performance and to identify the optimal classifier for use in further analysis and validation. All machine learning steps were conducted using the Weka toolkit [24]. While the 15 machine learning algorithms used in this analysis represented a diverse set well suited to the discrete nature of the data studied, we did not test many other algorithms common to machine learning including classification approaches that employ graphical models and Bayesian inference [25].

### Validating the Classifier

Although the 10 fold cross-validation steps served as an internal validation of classifier accuracy, we also tested the performance of the classifier using independent, age-matched ADI-R data from other families with autism whose data have been stored in the Simons Simplex Collection [26] (SSC) version 11 and in the Boston Autism Consortium collection (AC). The SSC data consisted of 1654 individuals classified with autism by the diagnostic standards of ADI-R and 5 that were found to be non-spectrum according to the Collaborative Programs of Excellence in Autism (CPEA) diagnostic algorithms established by Risi et al. [27]. The families in the SSC study were all simplex, i.e. only one child in the family with an ASD diagnosis. The AC set contained 322 individuals classified through the standard 2003 ADI-R as having autism and 12 classified as non-spectrum. Our objective with these independent resources was to determine if the classifier constructed from the AGRE dataset could accurately identify autism spectrum cases and non-spectrum controls.

### Exclusion of Questions

Prior to executing the machine learning algorithms on our data matrix, we removed questions from consideration if they contained a majority of exception codes indicating that the question could not be answered in the format requested. We also removed all ‘special isolated skills’ questions and optional questions with hand-written answers. A list of questions removed is available in Table S1.

### Balancing Classes through Simulation

Due to the limited number of non-spectrum controls and because machine learning algorithms maximize performance criteria that place equal weight on each data point without regard to class distinctions, we elected to simulate controls to increase the number of score sheets that would correspond to an ADI-R classification of non-spectrum. This enabled us to test whether the imbalance in the classes of autism and non-spectrum introduced biases that skewed downstream results. To create the simulated controls, we randomly sampled scores from the existing set individuals who did not meet the criteria for a classification of autism or autism spectrum in all three studies (N = 84) for all of the 93 items in the ADI-R. This guaranteed the simulated scores were drawn from the same distribution of observed scores. We repeated this process 1000 times to create artificial controls for use in secondary measurement of the specificity of the classifier. In addition, we simulated 1000 controls based on a complete collection of answers that would correspond to an official ADI-R classification of non-spectrum. The ADI-R algorithm sums scores within the three behavioral domains generating autism diagnosis when all three totals exceed specified minimum cutoffs, a value of 10 for social interaction, 8 if verbal or 7 if nonverbal for the communication and language domain, and 3 for restricted and stereotyped behaviors. We designed our simulation to randomly generate controls that would be close to but not exceed values for all three. This enabled us to explore a larger distribution of scores than available in our observed controls sample, and to examine scores that might represent behaviorally borderline subjects. Both simulated datasets were used to measure the specificity of the classifier and to evaluate potential biases stemming from class imbalance.

## Supporting Information

Table S1 **List of all the excluded questions from the ADI**-**R.** We removed questions from consideration if they contained a majority of exception codes indicating that the question could not be answered in the format requested. We also removed all ‘special isolated skills’ questions and optional questions with hand-written answers.
(PDF)
