# Supplementary material for: Use of Artificial Intelligence to Shorten the Behavioral Diagnosis of Autism
Source: PLoS One. 2012 Aug 27;7(8):e43855. doi: 10.1371/journal.pone.0043855 (PMC3428277; doi:10.1371/journal.pone.0043855)
Supplement: Table S1 — List of all the excluded questions from the ADI-R. We removed questions from consideration if they contained a majority of exception codes indicating that the question could not be answered in the format requested. We also removed all ‘special isolated skills’ questions and optional questions with hand-written answers. (PDF) [file pone.0043855.s001.pdf]

Supplementary Table 1

List of all the excluded questions from the ADI-R. We removed questions from consideration if they contained a majority of exception codes indicating that the question could not be answered in the format requested. We also removed all ‘special isolated skills’ questions and optional questions with hand-written answers.

Question Number on ADI-R	Question Code	Question Subject	Reason for Exclusion
1-CMT	concern	Current concerns	Comment
1a-CMT	current_concerns _specifiy	Specific current concerns (examples)	Comment
2	acon	Onset of symptoms	Too many exclusion #s/can’t answer question in format requested
3-CMT	fsymc	First symptoms to arouse parental concern	Comment
3a-CMT	fsymnc_specify	Specific symptoms to first arouse parental concern (examples)	Comment
5	walk	First walked unaided	Too many exclusion #s/can’t answer question in format requested
5-CMT	walk_cmmt	First walked unaided comments	Comment
6	drydz	Acquisition of bladder control: daytime	Too many exclusion #s/can’t answer question in format requested
7	drynz	Acquisition of bladder control: nighttime	Too many exclusion #s/can’t answer question in format requested
8	bowel	Acquisition of bowel control	Too many exclusion #s/can’t answer question in format requested
8-CMT	bowel_cmt	Acquisition of bowel control comments	Comment
9	aword	Age of first single words	Too many exclusion #s/can’t answer question in format

			requested
9-CMT	aword_cmt	Age of first single words comment	Comment
10	aphrase	Age of first phrases (if ever used)	Too many exclusion #s/can't answer question in format requested
10-CMT	aphrase_cmt	Age of first phrases (if ever used) comments	Comment
11-CMT	loslang_cmt	Loss of language after acquisition comment	Comment
12-CMT	levlang_cmt	Level of communicative language before loss comment	Comment
13-CMT	lossp_cmt	Loss of spontaneous use of at least five meaningful words comment	Comment
14-CMT	losword_cmt	Loss of communicative intent comment	Comment
15-CMT	losssyn_cmt	Loss of syntactical skills (grammar) comment	Comment
16-CMT	lossart_cmt	Loss of articulation (pronunciation)	Comment
17	aloslan	Age when main loss of language skills first apparent	Too many exclusion #s/can't answer question in format requested
17-CMT	aloslan_cmt	Age when main loss of language skills first apparent comment	Comment
18-CMT	losphys_cmt	Association of loss of language with physical illness comment	Comment
19	losdurn	Duration of loss of language skills	Too many exclusion #s/can't answer question in format requested
19-CMT	losdurn_cmt	Duration of loss of language skills comment	Comment
20-CMT	elskil_cmt	Loss of skills (for at least 3 months) comment	Comment
21-CMT	elhand_cmt	Purposive hand movements (ability to grip/hold objects) comment	Comment
22-CMT	elmotr_cmt	Motor skills (posture, gait, coordination) comment	Comment
23-CMT	losshlp_cmt	Self-help skills (feeding, dress, using the bathroom etc.) comment	Comment
24-CMT	elplay_cmt	Constructive or imaginative play (puzzles, games, make-believe etc.) comment	Comment
25-CMT	elsoc_cmt	Social engagement and responsiveness (social relatedness, interest, and involvement) comment	Comment

26	ageloss	Age when main loss of skill first apparent	Too many exclusion #s/can't answer question in format requested
27-CMT	elskph_cmt	Association of loss of skills with physical illness comment	Comment
28	durdet	Duration of loss of skills	Too many exclusion #s/can't answer question in format requested
28-CMT	durdet_cmt	Duration of loss of skills comment	Comment
36-CMT	inappq_cmt	Inappropriate questions or statements comment	Comment
37-CMT	pron_cmt	Pronominal reversal comment	Comment
38-CMT	neoid_cmt	Neologisms/idiosyncratic language comment	Comment
39-CMT	verrit_cmt	Verbal rituals comments	Comment
40-CMT	inr_cmt	Intonation/volume/rhythm/rate comment	Comment
41-CMT	speech_cmt	Current communicative speech comment	Comment
43-CMT	nod_cmt	Nodding comment	Comment
44-CMT	hshake_cmt	Head shaking comment	Comment
46-CMT	attinst_cmt	Attention to voice comment	Comment
47-CMT	imit_cmt	Spontaneous imitation of actions comment	Comment
57-CMT	rfacex_cmt	Range of facial expressions used to communicate comment	Comment
58-CMT	einapf_cmt	Inappropriate facial expressions comment	Comment
No assigned question number	favtoycmt	Favorite activities/toys	Comment
87	judgage	Interviewer's judgment on age when developmental abnormalities probably first evident	Not multiple choice
88	cvisspz	Current visuospatial ability (in puzzles, jigsaws, shapes, patterns, etc.)	Low response
88a	evisspz	Visuospatial ability ever (in puzzles, jigsaws, shapes, patterns, etc.)	Low response
89	cmemz	Current memory skill (accurate memory for detail, as of dates or timetables)	Low response
89a	ememz	Memory skill ever (accurate memory for detail, as of dates or timetables)	Low response
90	cmusicz	Current musical ability (recognition, composition, absolute pitch, or	Low response

		performance)	
90a	emusicz	Musical ability ever (recognition, composition, absolute pitch, or performance)	Low response
91	cdrawz	Current drawing skill (unusually skilled use of perspective or creative approach)	Low response
91a	edrawz	Drawing skill ever (unusually skilled use of perspective or creative approach)	Low response
92	creadz	Current reading ability (e.g., early sight reading)	Low response
92a	ereadz	Reading ability ever (e.g., early sight reading)	Low response
93	ccompuz	Current computational ability (e.g., mental arithmetic)	Low response
93a	ecompuz	Computational ability ever (e.g., mental arithmetic)	Low response
